# Investigating the use of sensory information to detect and track prey by the Sunda pangolin (*Manis javanica*) with conservation in mind

**DOI:** 10.1038/s41598-020-65898-x

**Published:** 2020-06-17

**Authors:** Joshua D. DiPaola, Marnoch Yindee, Joshua M. Plotnik

**Affiliations:** 1 Animal Behavior and Conservation Program, Department of Psychology, Hunter College, City University of New York, 695 Park Avenue, Room 611N, New York, NY 10065 USA; 2 Livestock and Wildlife Hospital, Faculty of Veterinary Science, Mahidol University, 199 Moo 9, Highway No. 323, Sai Yok, Kanchanaburi, 71150 Thailand; 3 Psychology Program, The Graduate Center, City University of New York, 365 Fifth Avenue, New York, NY 10016 USA; 4 Present Address: Akkharatchakumari Veterinary College, Walailak University, 222 Thaiburi, Thasala, Nakhon Si Thammarat 80161 Thailand

**Keywords:** Behavioural ecology, Conservation biology, Psychology, Animal behaviour

## Abstract

Pangolins are of conservation concern as one of the most heavily poached, yet least understood mammals. The Sunda pangolin (*Manis javanica*) in particular is a critically endangered species. Here, we investigate the behaviour of these pangolins, for the first time, using a battery of cognitive tasks based on a manipulation of available sensory information. In an object-choice task in which only one of two containers was baited with food, the pangolins were able to find the food with olfactory information alone (N = 2), but not with visual or acoustic information alone (N = 1). The single subject tested on all three domains was further tested on how he used smell to find food by providing him with an opportunity to find it from a controlled distance or by using scent trails as a guide. The results suggest that our subject may have the capacity to exploit scent trails left by prey which can be tracked to a final source, though we found no evidence to suggest that he had the ability to initiate hunts based on distant prey odors. Despite the small sample size, this is the first controlled experiment to investigate pangolin foraging behaviour and cognition, which may have implications for the future protection of pangolin habitat based on the location of prey species.

## Introduction

One of the most important confounds in the design of comparative cognition research centers on the question of ecological validity. Drawing cognitive comparisons across evolutionarily distant taxa requires a basic understanding of an animal’s *umwelt*, with particular attention to how behaviours such as foraging and problem-solving are affected by sensation. For instance, while developing puzzles that require visual access and manipulation with tools makes ecological sense for animals like chimpanzees^1–3^ and corvids^4,5^, apparatuses that provide olfactory information and can be manipulated using an animal’s own body parts are more relevant for elephants^6–8^ and dogs^9,10^. Understanding the sensory perspective of a particular species is even more critical when designing behavioural or cognitive tests for animals that have rarely, if ever been studied previously. Investigations of the proximate, cognitive mechanisms that underlie an animal’s decision-making processes can have important implications both from a theoretical (i.e., for understanding how cognition evolves across species to help an animal forage, interact with conspecifics, and avoid predation, for example) and an applied conservation perspective. For the latter, there is a growing need for conservationists developing endangered species strategies to collaborate with ecologists, biologists and psychologists in order to gain a greater understanding of animal behaviour and cognition.

To this end, the experimental object-choice task can be used to better understand the importance of particular sensory modalities in an animal’s foraging behaviour. This task typically involves the presentation of two or more options where only one is baited with food. The experimenter can control how much or how little sensory information is available to the animal when it investigates and subsequently makes a choice between the provided options. This task has been used to investigate different capacities, including cue-following in the visual (e.g.^11–13^), acoustic (e.g.^6,14,15^), and olfactory (e.g.^6,7^) domains. It is a simple paradigm that can be reliably controlled, which makes it well-suited for testing the sensory perspectives of a highly understudied, nocturnal mammal.

The pangolin is unique in that it is one of the world’s most endangered and widely trafficked animals^16–21^, yet we know little about its behaviour or ecology^17,22,24–26^. The limited scientific data on pangolins are concerning considering that all eight species are threatened with extinction^16^. The primary threats to conserving wild pangolins are unsustainable levels of poaching for traditional medicine and bushmeat, and habitat loss^25–28^. Much of the literature on pangolins details the degree to which the illegal wildlife trade continues to drastically affect their population numbers^17–21^, with limited investigation thus far of how pangolins behave in their natural environments^17,22,24–26,29^. Conservation concern for pangolins is further complicated by the difficulty of studying them in the wild^17,25,29–31^, as their reduced numbers provide fewer sampling opportunities and our lack of understanding regarding their ecology impairs our ability to locate those that remain. Opportunities to study and conserve pangolins *ex-situ* are also rare, given that there is a particularly high mortality rate associated with maintaining them in captivity^17,31–37^. Pangolins often die in captivity as a result of improper diets^17,35,38,40^ and the difficulty of rehabilitating them after they have been exposed to the physical traumas of illegal poaching and smuggling^32,39–42^. Given the challenges of studying pangolins both in captivity and the wild, any opportunity to study their behaviour could have a significant, positive impact on both captive welfare and wild conservation efforts.

Pangolins are unique mammals with dorsal scaling, no teeth, prehensile tails, and a long tongue that protrudes during foraging^24,43,44^. Although there are eight species with distinct characteristics within the pangolin taxon, most are primarily nocturnal^24,44^, and prey on ants and termites^22,23,38,44^. Research on pangolin sensory perception is remarkably lacking. Studies on the brain anatomy of African tree pangolins (*Phataginus tricuspis*, formerly *Manis tricuspis*) suggest they have enlarged olfactory bulbs relative to other mammals, but non-remarkable visual and auditory systems^28,45–47^. Behavioural anecdotes of pangolins using their elongated snouts and tongues to forage seem to support the idea that they rely on olfaction and somatosensory information to find food^24,36,45,46,48^. Although pangolin vision is considered relatively poor^24,36,49^, recent research on the anatomy of the pangolin eye suggests it may be well-adapted for the nocturnal environment^50^. Research on pangolin audition is particularly sparse, with mixed reports on the quality of their hearing^24,36^, and no direct evidence of how they might integrate acoustic information into their general behavioural ecology^47^.

The current study specifically investigates the foraging behaviour of the semi-arboreal Sunda pangolin (*Manis javanica*)^24,26,33^, one of the most threatened mammals on the planet^51^. Here, we use several experimental paradigms to investigate the pangolin’s use of vision, hearing, and smell to locate food. In Phase I of this study, we tested the pangolin’s ability to locate food in a two object-choice task by controlling for two of these three sensory modalities and allowing the animal to investigate and choose, using the remaining sensory information, which one of two containers held food. In other words, we blocked two types of sensory information coming from the food source and thus from being perceived by the pangolin in order to assess whether it was able to use the remaining information alone to find the food. In Phase II, we developed two novel olfaction tasks to investigate whether or not the pangolin was using distant or proximal prey odors to find food. Although there is little known about how pangolins locate food in their environments, our own observations of the pangolin’s use of its pronounced snout led us to predict that olfaction would be their dominant sensory modality when foraging.

## Methods

### Subjects

This study was conducted between January–March and June–July, 2018 with two wild-born, sexually mature Sunda pangolins (M = 1, F = 1) housed at the Livestock and Wildlife Hospital of the Faculty of Veterinary Science, Mahidol University, Kanchanaburi, Thailand. Permission to conduct this research was granted by the director of the hospital (co-author, M.Y.), in whose care these pangolins were under. Our initial sample included three subjects (M = 2, F = 1), however, one pangolin showed no interest in the testing apparatus while being habituated to the research materials and thus, no data were collected on this subject. The two subjects that participated in the study were tested directly within their enclosures to minimize stress associated with handling. The pangolins’ housing consisted of semi-enclosed rooms in an outdoor barn at the veterinary hospital, and the animals had access to water, shelter and climbing structures.

The pangolins had strict diets developed by the veterinary staff to maintain their health. They were each given a predetermined quantity of dead weaver ants (*Oecophylla smaragdina*) once per day (in the evening), with occasional supplements of live ants. Due to the predetermined, singular feeding period as well as the relatively fragile nature of the pangolins’ health in captivity, we did not withhold or reduce their diet to increase their motivation levels for testing. The Hunter College Institutional Animal Care and Use Committee (protocol # JP-Pangolin-11/17) reviewed and approved this research, and the methods were carried out in accordance with the relevant guidelines and regulations.

### Procedure

**Figure 1:**
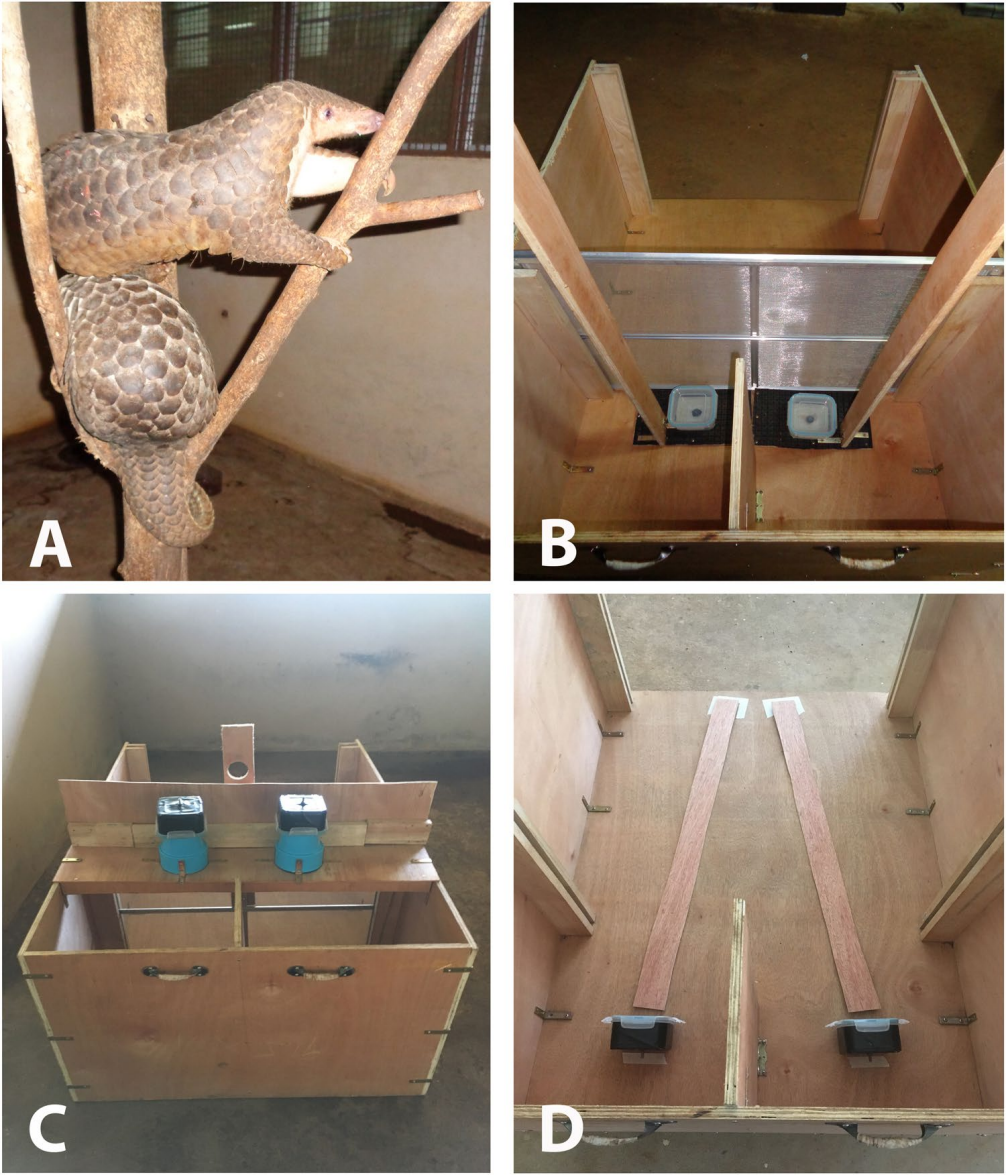
Images of the pangolins and experimental set-ups for both phases of testing. (**A**) Two Sunda pangolins in an artificial tree inside their testing area. (**B**) Experimental setup used for all Phase I conditions (the containers in this photograph are controlled for a ‘visual’ condition trial). The view is from behind the testing apparatus. Once the pangolin enters the chamber (from the top of the image), the metal mesh screen is removed and the pangolin can investigate the two containers. The containers are then pulled to the back of the chamber, at which point the pangolin can make a choice and the lid of the chosen container is removed. (**C**) Experimental setup for ‘olfactory distance’ testing in Phase II. Similar to Phase I, the pangolin entered the chamber (from the top of the image), the screen was removed, and the pangolin could investigate the two containers, which were positioned on top of PVC pipes above the pangolin’s head. This photograph represents a 15-cm training trial. (**D**) Experimental setup for ‘scent trail’ testing in Phase II. The pangolin could again enter the chamber but there was no screen to remove; the pangolin could follow the scent on either of two wooden planks that led to a baited (‘food’ plank) or unbaited (‘water’ or ‘mint’ plank) container. This image does not include the cloth tape applied to the planks prior to testing. All photos by J.D.D.

These animals were first tested in Phase I of this study on their ability to locate food, a sample of ants, across foraging tasks within three sensory domains: visual, acoustic, and olfactory (see Plotnik *et al*.^6^ for similar procedures on which the current research is based). Because Sunda pangolins are nocturnal^24,26,33,48,52^, all testing was conducted between 1800–0200 hrs. For initial sensory testing, the pangolins were presented with a basic object-choice task in which two hard plastic 10 cm × 10 cm × 6 cm containers, one containing food the other not, were presented inside a wooden testing chamber. Each container rested flat on top of its own sliding wooden platform, which had a 107 cm vertical wooden handle attached so that the experimenter could manipulate the position of the containers throughout a trial without physically entering the testing chamber (Fig. 1). Because the pangolins were wild-caught and had limited experience with novel objects prior to testing, we first habituated the animals to the paradigm by allowing them to retrieve food from a single baited container without a lid. We then gave them an opportunity to search two open containers, with only one baited, and then repeated this inside the testing chamber. Once they voluntarily entered the chamber and searched the two containers consistently, testing began on the two-object choice task. For each trial of testing in Phase I, the pangolin could first approach two locked containers (with lids) placed adjacent to each other on their respective wooden platforms. During this time, the pangolin could directly touch and investigate both containers. After 10 s, the experimenter used the vertical handles to pull the two containers to the back of the chamber where a short, 40 cm partition separated the two containers. The purpose of this partition was to isolate each container to make the decision-making process as clear as possible to both the subject and experimenter. The pangolin was then given 30 s to make a choice. The criterion for ‘choice’ required that the pangolin pass the partition and touch the container with its snout or claws. This criterion for ‘choice’ was used for all conditions except Phase II ‘olfactory distance’ testing (see below for details on this condition). The experimenter then manually unlocked the lid of the chosen container and withdrew the alternative by lifting it out of the apparatus using the vertical handle. If a pangolin failed to make a choice in the time allotted, the trial was reset. All experimental testing was voluntary, with the pangolins being allowed to enter and exit the testing area at will as soon as the testing apparatus was rebaited after each trial. Rebaiting of containers happened out of view of the pangolins between trials, and metal mesh screens were used to temporarily block off the apparatus while it was rebaited. Because we did not want to handle the pangolins or restrict their movement within their enclosures, there was no specific inter-trial interval (ITI) established for testing.

In Phase I of testing, the pangolins were presented with three conditions that each provided sensory information about the presented food from a single modality (see Supplementary Video S1). The containers were manipulated so that they were either: a) opaque, held 35 g of dead ants and were locked with perforated lids (so that the pangolins could smell but not hear or see the ants – the ‘olfactory’ condition), b) transparent with solid lids and 35 g of dead ants (so that the pangolin could see but not hear or smell the ants – the ‘visual’ condition), or c) opaque with solid lids and live ants (so the pangolin could hear but not see or smell the ants – the ‘acoustic’ condition). Containers and lids were made opaque using black duct tape applied on the inside. In this last condition, the average weight of the live ant nests (the ants plus the leafy substrate from their environment) in the closed baited container was 45 g. In addition, even though the pangolins are nocturnal, the ‘visual’ condition was conducted either in the presence or absence of an artificial light to investigate whether visual light made any difference in the pangolin’s ability to locate the food. Control trials utilized opaque containers, solid lids and 35 g of dead ants (so that pangolins had no access to sensory information about the ants), and allowed us to investigate whether the pangolins were using cues independent of the food present – e.g., inadvertent experimenter cuing – to make a choice. The 35-g amount of dead ants for each trial within a non-acoustic condition was determined based on the average weight of a live ants’ nest minus the substrate, as well as a relatively equal distribution of the pangolin’s daily diet across an evening’s test trials as determined by the hospital’s veterinary staff. Subjects were given the opportunity to participate in one testing session per day across both Phase I and Phase II of this study. Testing was conducted on consecutive days unless the subject failed to voluntarily participate, in which case testing was delayed until the next day. Phase I conditions were presented in the following order: olfactory, visual no-light, visual light, acoustic untreated, and acoustic treated. The ‘olfactory’ condition consisted of four sessions of 12 trials (eight test and four control). The ‘visual’ condition consisted of two parts: four sessions of 12 trials (eight test and four control) presented in the absence of an artificial light source (no-light), followed by an equal number of trials in the presence of light. In the first three sessions of the ‘acoustic’ condition, live weaver ants were collected from the environment and immediately presented to the pangolin in the same containers for testing (‘acoustic untreated’ - three sessions of 12 trials (eight test and four control)). It was very difficult to move the ants between containers once they had been collected due to their high level of aggression and fast movement. Because we realized later that the ants may have left olfactory cues on the exteriors of the containers during collection, we ran an additional three sessions of 12 trials in which we meticulously cleaned the outside of the containers between ant collection and presentation to the pangolin using a mild soap and water solution (‘acoustic treated’). In all types of condition, containers were cleaned between sessions. In the ‘acoustic’ condition, the ‘baited’ container remained unchanged throughout a session (due to the impracticality of moving a live ant colony easily between trials), however, the ‘baited’ container was placed on each side of the apparatus an equal number of times. In this ‘acoustic’ condition, the pangolin was rewarded with a separate quantity of dead ants if it correctly chose the container with live ants, to avoid having to open and rebait the live ant container. Because the ‘olfactory’ and ‘visual’ conditions utilized dead ants throughout, both containers were ‘baited’ or ‘unbaited’ an equal number of times within each session to avoid any confound of residual odor (e.g., from previously baited food or the pangolin’s own scent). All trials across the ‘olfactory’, ‘visual’, and ‘acoustic’ conditions of phase I were pseudo-randomised within a session so that each session consisted of an equal number of trials of left and right correct choices (based on the side of the chamber in which a container was placed), no one side was baited with food more than three consecutive times in a row, and the first three trials for each session across all conditions were always tests rather than controls to avoid a frustration effect.

The female pangolin, ‘Betty,’ was only tested on a portion of her first condition, the initial ‘olfactory’ condition (Table 1), because we noticed a significant decrease in both her nightly food intake and her interest in entering the experimental apparatus as testing progressed. Because we did not want to negatively impact her welfare state, she was not subjected to any further testing. The male pangolin, ‘Pluto’, was tested on all of the conditions as he remained highly motivated throughout testing.

**Table 1:** Raw data for both subjects (‘Pluto’, the male, and ‘Betty’, the female) across all test and control conditions in Phase I and II. The ratios provided represent the number of trials in which the pangolin selected the correct, baited container (numerator) over the total number of trials for each respective condition (denominator). The top of each column provides an abbreviation of the condition tested with the type of trial listed below. Control trials are represented by “C”. Test trials are represented by “T”. In Phase I, the olfactory condition was comprised of only one type of test trial, while visual and acoustic conditions included two separate types each. In Phase II, the ‘olfactory distance’ condition was comprised of three types of test trials based on the distance at which the stimuli were presented, and are delineated in the table by this distance in centimeters (cm). There were two types of test trials in the ‘scent trail’ condition. ‘Scent trail’ test trials which provided a food vs. mint scent pair are represented by “F vs M”. Test trials which provided a food vs. water scent pair are represented by “F vs W”. Ratios that are bolded and marked with an asterisk indicate that the pangolin performed significantly better than chance (P < 0.001) (*binomial test, two-tailed). See the methods section for details on each condition across the two phases.

*Phase I*	Olfaction		Visual – Light		Visual – No Light		Acoustic – Untreated		Acoustic – Treated	
	*T*	*C*	*T*	*C*	*T*	*C*	*T*	*C*	*T*	*C*
Pluto ♂	27/32*	6/16	19/32	9/16	17/32	9/16	15/24	8/12	11/24	6/12
Betty ♀	20/20*	1/8								
***Phase II***	**Olfactory Distance**				**Scent Trail**					
	***T-30cm***	***T-60cm***	***T-90cm***	***C***	***T-F vs M***	***T-F vs W***	***C***			
Pluto ♂	10/20	10/20	8/20	8/20	20/30	20/30	9/20			

In Phase II of the study, the male pangolin was tested on two different olfactory conditions. The ‘olfactory distance’ condition specifically aimed to investigate whether pangolins may detect their prey or initiate a hunt based on distant olfactory cues (see Supplementary Video S2). This condition was similar to the Phase I olfactory condition with the following exceptions. First, in ‘olfactory distance’ trials, the pangolin could not physically touch either container while he first investigated them. Instead, he could only smell, but not touch the containers from a distance of 30, 60 or 90 cm. Second, the pangolin was given 15 s to investigate both containers in ‘olfactory distance’ testing compared to the 10 s used for the olfactory condition in Phase I. The investigation time was increased for ‘olfactory distance’ trials to ensure that the pangolin had sufficient time to navigate the additional spatial components of this condition (details below). Third, the containers were no longer presented to the pangolin for investigation on the floor of the apparatus in ‘olfactory distance’. They were now positioned overhead on a vertical plane to best mimic what the pangolin might spatially encounter in an arboreal hunt; he would now need to smell upwards in order to gain access to the food’s olfactory information. The containers were placed porous lid-side down on top of a solid, PVC tube cut to the predetermined length/distance and set on top of a wooden plank overhanging the testing chamber (Fig. 1). Holes were cut into this plank so that the pangolin could smell into the tubes (and thus the distant containers) above it.

In any given training or test trial of the ‘olfactory distance’ condition, after the pangolin entered the testing chamber, he had 15 s to investigate the two sides. The containers were then placed on the floor of the chamber so the pangolin could choose one. The pangolin then had 30 s to pass the 40-cm partition separating the two containers, at which point the experimenter unlocked and removed the chosen container’s lid, while the other container was removed (see Supplementary Video S2). This early designation of the pangolin’s choice (i.e., before he made contact with a container) was used to minimize his opportunity to make a decision using olfactory information from within the containers once they were placed on the floor. The food reward used in this condition consisted of 35 g of dead ants. Since this was the pangolin’s first experience with testing on a vertical plane, he was first trained with the containers placed directly overhead on the wooden plank without the added distance created by the PVC tubes. The pangolin reached the criterion of 80% correct in a single session of eight trials (i.e., seven out of eight trials correct) within his first training session. Subsequently, he was trained on a distance of 15 cm to ensure he understood the task with all of the apparatus components in place, reaching the same criterion in his third session. Following completion of training, the pangolin participated in the test condition consisting of ten total sessions. Each testing session included six test trials (two trials of each distance, randomised) and two control trials, for a total of eight trials per session. Control trials across all ‘olfactory distance’ testing were conducted at the shortest distance (30 cm) using the same control containers from Phase I. As in Phase I, in the ‘olfactory distance’ condition, once the containers were placed on the PVC by the experimenter, the metal mesh screen was removed so the pangolin could enter the apparatus. Again, no inter-trial interval (ITI) was set to avoid having to handle the pangolin or restrict his movements, however, he never entered the apparatus sooner than 5 s after the containers were placed on the PVC (it usually took considerably longer), and he always had 15 s to investigate them before they were removed and placed on the ground. Thus, at least 20 s passed between setting a trial and the pangolin having to make a choice.

The ‘scent trail’ condition aimed to investigate whether pangolins could use scent trails to track their prey (see Supplementary Video S3). Due to the pangolin’s natural tendency to smell as it moved through the wooden chamber, no training was needed for this condition. Test trials made use of two wooden planks on each of which a strip of cloth tape was applied to prevent the smeared liquid from running. A different scent was smeared on each tape strip using a sponge (approximately 5 mL of scent was applied per strip, per trial). One of two stimulus containers (one baited and one unbaited) was placed at the end of each plank, on its side and with the locked lid facing the pangolin. Once the animal voluntarily walked into the testing chamber, he had 30 s in which to make a choice. As soon as he reached one of the two containers, the lid was unlocked and removed. For all test trials, the food (F) scented trail always lead to the baited container. This scent was extracted by taking dead weaver ants and pressing them within the barrel of a syringe, producing a concentrated liquid extract. In one type of test trial, the scent of ants (F) was applied to one trail while plain tap water (W) was applied to the non-food bearing trail (F vs. W). The W trail acted as a control for a scent ‘smear’ by providing comparable characteristics of the ant ‘smear’ without the odor of prey. For the other type of test trial, the scent of ants (F) was applied to one trail while the non-prey odor of mint (M) was applied to the alternative trail (F vs. M). The mint odor was produced by gathering wild mint leaves, dicing them and soaking them in warm water 30 min prior to testing. The resulting extract (devoid of the leaves) was smeared on its own plank. This condition investigated whether the smell of a non-prey, naturally occurring odor was distracting or potentially more interesting than prey-derived odors.

Phase I control containers (i.e., opaque with solid lids) were used as the stimulus containers in the test trials for this condition as we wanted the subject to make olfactory decisions based on the trail scent without being influenced by the odor of the food inside the container. ‘Scent trail’ control trials followed the same procedure as all prior testing in this condition except that the trail strips used were devoid of any smear. These trials again ensured the pangolins were not using any cues besides those which we intentionally provided for them to find the food. This condition consisted of ten sessions, with each session consisting of two control trials and six test trials (three F vs. M and three F vs. W trials). Prior to each trial, scents were reapplied to their respective planks. Phase II pseudo-randomisation procedures were identical to that of Phase I except that only the first two trials in a Phase II session were always test trials; the remaining trials within a session contained a randomised order of test and control trials.

All experimental conditions were recorded using the built-in infrared NightShot capabilities on a SONY FDR-AX100 (see Supplementary Videos S1-S3). Trials were coded for the pangolin’s container choice in real-time by J.D.D.

## Results

Both subjects selected the baited container significantly more often than chance in the olfactory condition of Phase I (P < 0.001 for both pangolins, two-tailed binomial test). The olfaction results for the female, however, reflect only partial completion for this condition. She did not participate in any other testing. The male did not perform significantly better than chance in any other condition in Phase I or in the ‘olfactory distance’ condition in Phase II (Table 1).

In the ‘scent trail’ condition, the male chose the food-scented trail significantly more often than chance across all 60 trials (40/60 trials, P = 0.013, two-tailed binomial test). Although he chose the food-scented trail more often in both types of ‘scent trail’ test trial (the food vs. mint and food vs. water conditions) – 66.7% in both – the results were not statistically significant when analysed separately (20/30 trials each, see Table 1).

## Discussion

In this study, two pangolins (M = 1, F = 1) demonstrated a capacity for finding food using olfactory information alone. One pangolin’s performance (M = 1) on the additional olfactory foraging tasks suggests he may have been using scent trails to track prey rather than olfactory information at a distance directly from the source. In addition, this pangolin was unable to use visual or acoustic information to find food when this was the only sensory information about the food’s location available to him. The two pangolins’ poor performance on control trials confirmed that they did not use any other inadvertent cuing from the experimenter to find the food.

In order to discuss and interpret these results, we recognise the small sample size is a significant limitation. In particular, most of our results are based on the performance of a single pangolin. Further testing with a larger sample size that focuses on different types of visual and acoustic stimuli in a foraging context would be illuminating, and would allow for extrapolation of results to a larger population within and potentially across pangolin species. Without access to a larger sample size and the opportunity to test multiple subjects with a randomised order of conditions, we also cannot exclude the possibility that the data from our one subject were influenced by a possible order effect. Though we did not find any evidence to suggest this, it is also possible that our subject had sensory impairments which could have impacted our results. However, due to the extreme difficulty of testing pangolins in controlled settings and the Sunda pangolin’s status as a critically endangered mammal on the verge of extinction, we present these results as a case study. We feel it is important to hypothesise about how a single pangolin’s performance may help inform our understanding of pangolin behavioural ecology and cognition.

The pangolin’s performance on the visual condition, for example, makes sense considering the pangolin’s morphology and known ecology. The Sunda pangolin is a predator with relatively small eyes that forages on small insects at night^24,26,48^, and thus vision may not be as relevant to its foraging strategy as olfaction. It is also possible that our use of dead ants as the rewarding stimulus was not ecologically salient enough for the pangolin to locate the food visually. In addition, the pangolin in this study did not demonstrate an ability to locate its food based on the acoustic cues provided by live ants. Although we included leaves as a substrate in order to increase the sound produced by the ants inside the container, the plastic may have muted the acoustics enough that the pangolin could not differentiate between the baited and unbaited containers. However, from an ecological perspective, if pangolins do use their hearing to hunt insects in the wild, they would have to detect their acoustic signatures from varying distances and amid ambient noises within their natural habitats. They would also need to be able to hear their prey through tunnels in the ground, dense dirt mounds, or high within tree nests. Thus, at close proximity, if the pangolin in our study used acoustic information to locate prey, he should have been able to detect it even behind the artificial plastic barrier. Our own experience with the three pangolins suggests they do react to loud noises, and may adjust their tail posture based on the substrate on which they are walking. This behaviour may indicate that pangolins adjust their locomotion and their tail’s position to minimize the noise they make in the wild. Thus, although the pangolins may not use acoustic information when locating prey, it is likely they use it in predator detection or avoidance. Plotnik *et al*.^6^, for instance, found that Asian elephants did not use auditory cues to find food in a similar object-choice task, even though elephants are well-known for their complex acoustic repertoires (e.g.^53–55^). Like for elephants, the “sound food makes” may not be important for the pangolins to find it.

Considering the coevolution that can take place between predatory and prey (e.g.^56–58^), it might make more sense that the predatory habits of the pangolin would have evolved to exploit the more obvious chemosensory signatures generated by their prey. Ants and termites rely heavily on the transmission of odors and pheromones to communicate amongst themselves^59–62^. Thus, a keen sense of smell would allow the pangolin to efficiently exploit the abundant availability of these olfactory signatures. From a foraging perspective, it seems likely that pangolins would prioritize olfactory information when searching for prey.

While it is unknown if it is pheromones or other prey chemicals (such as odor from larvae) that influence the pangolin’s navigation in a hunt, we tried to understand how olfaction was functionally employed by pangolins across different ecological scenarios. Since the Sunda pangolin has been noted for its semi-arboreal foraging behaviour^26,48^, ‘olfactory distance’ testing was conducted on a vertical plane to best replicate a foraging situation that a wild pangolin might encounter as it walks through a forest in search of arboreal prey. In the absence of nearby environmental cues, we found that our subject was either not sufficiently motivated by the distant prey odor offered, or was unable to locate it based on the olfactory information available. Another possible explanation for our results in ‘olfactory distance’ testing was our inability to use a specific, longer inter-trial interval (ITI) in this condition to ensure that the odor molecules had sufficient time to make their way from the container at the top of the PVC down to the ground, thus creating an odor concentration gradient sufficient enough to be detected by the pangolin. Due to the danger of the experimenter negatively impacting the pangolin’s health by excessive handling or spending too much time inside his enclosure, we could not enact a longer ITI. Thus, the minimum time between trials plus the time the pangolin had to investigate was at least 20 seconds. The food amount used in each trial, 35 g of ants, was determined based on the average weight of an ant nest minus substrate, and thus this should also have enhanced the pangolin’s capacity for finding them in a short period when the smell was confined within a tube and that smell was of a sufficient, ecologically relevant concentration. Another potential confound in all olfactory conditions was the fact that we could not completely remove residual odor between trials, and thus the pangolin’s choices may have been confused by olfactory information from prior trials. However, the pangolins’ success on Phase I olfaction trials and Phase II ‘scent trail’ trials suggests residual odor was likely not enough to overcome the pangolins’ interest in odors emanating from a present food source, and thus the one pangolin’s poor performance on ‘olfactory distance’ was likely not due to residual odor. In addition, the pseudo-randomisation of distance trials and the placement of different PVC tube lengths also meant that residual odor was regularly dispersed when tubes were removed and replaced, making it an unreliable source of information about food location. Finally, if residual odor was a confound, we would expect the pangolin’s performance on control trials to be influenced by the location of food in the test trials immediately preceding them. In a post-hoc review of these trials in ‘olfactory distance’ testing, we found that of the 16 control trials that immediately followed a test trial, Pluto the pangolin chose the same location in the control trial that had the baited container in the immediately preceding test trial only six of 16 times (two-tailed binomial, P = 0.454). This suggests he was not using residual odor to find his food. In studies of elephants, another highly olfactory animal, they too did not seem to use residual odor to guide their choices about food^6,7^.

Despite the pangolin’s poor performance on the ‘olfactory distance’ condition, it seems unlikely that a predator with a developed olfactory system^24,26,36,45,46,48^ would be unable to detect its prey from the distances we investigated. Scent trails would be most useful once they are found, but detection of distant odors may offer valuable information about directionality when there are no immediate environmental cues available. Overall, the performance of our subject in the ‘scent trail’ condition suggests that the proximate environmental cues offered by scent trails may offer a more efficient method to localize live, mobile prey. Our results were only significant in this condition when viewed in aggregate (i.e., across all ‘scent trail’ trials but not within food vs. mint or food vs. water trials). However, the similar percentage correct on each of these trial types (66.7%) suggests that the pangolin likely could differentiate between the food and the non-food odor trails and followed the former. In fact, the anatomy of the pangolin may be conducive to following scent trails, as it has a low-hanging head and elongated nose that slopes towards the ground during terrestrial locomotion. The reliance on terrestrial scent trails, however, is somewhat confounding considering the semi-arboreal ecology of Sunda pangolins^25,26,33,48^ and we believe further testing is needed to understand whether the value of foraging cues changes as pangolins move from the forest floor into the trees. For instance, perhaps pangolins prioritize different sensory information as they pursue prey across different spatial planes, or they may follow olfactory information continuously as they track their food’s movements.

## Conclusion

It is not always clear how the study of animal cognition fits into improving welfare and conservation in practice^63,64^. The goal of this research was to identify the pangolin’s primary sensory modality in foraging behaviour, and then how that modality (olfaction) may be used to track prey. Although this is only one study with a small sample size, research on the behaviour and cognition of animals like pangolins may have important applications for *ex-situ* captive management^17,35,39,65^. Considering the high mortality rates of pangolins in captivity due to artificial diets and a lack of knowledge about their ecology^17,31–39^, a greater understanding of the pangolin’s natural behaviour (including foraging) is crucial for improving the welfare of these animals when reliant on human care.

Investigating pangolin behavioural ecology and cognition is also relevant to their conservation in the wild^17,22,23,25^. It is possible, for instance, that pangolins not only detect and value the scent of their prey, but also associated odors linked to it, such as the aroma of specific flora typically inhabited by their prey. In addition, although our study did not investigate ecologically relevant non-food odors in detail, it is possible that pangolins may prefer areas of their habitat which are devoid of predatory odors, while the presence of conspecific odors may influence their movement patterns based on whether the olfactory information comes from competitors or mates. Our study, while limited, could complement future research on pangolin foraging behaviour to help create more efficient conservation protocols that focus on protecting pangolin food resources and habitat rather than on locating elusive, individual animals. A broader perspective on how pangolins use sensory information to forage and navigate through their environment could thus highlight key conservation points to protect not only pangolins, but the more discrete resources which they value as well.

Finally, the COVID-19 pandemic highlights a crucial need to look beyond the charisma of endangered species when deciding which animals deserve the field of conservation’s attention. It is vital that we work to better understand wildlife behaviour and ecology through research across scientific disciplines, even when focal species are difficult to study or lack popular or political attention. Although it is still unknown whether the pangolin acted as a vector for the coronavirus between wildlife and humans^66,67^, what is clear is that the illegal wildlife trade and the increasing physical contact between wildlife and humans pose an existential threat not only to biodiversity in general, but also to our own existence as a species.

## Supplementary information

Supplementary Information.

Supplementary Information.

Supplementary Information.

Supplementary Information.

## Data Availability

All of the raw data that support the findings of this study are included in Table 1.
