# Supplementary material for: Investigating the use of sensory information to detect and track prey by the Sunda pangolin (Manis javanica) with conservation in mind
Source: Sci Rep. 2020 Jun 17;10:9787. doi: 10.1038/s41598-020-65898-x (PMC7300028; doi:10.1038/s41598-020-65898-x)
Supplement: Supplementary file 1 — Supplementary Information. [file 41598_2020_65898_MOESM1_ESM.pdf]

**Supplementary Information**

# Investigating the use of sensory information to detect and track prey by the Sunda pangolin (*Manis javanica*) with conservation in mind

Joshua D. DiPaola[1], Marnoch Yindee[2*], Joshua M. Plotnik[1,3]

**Author Affiliations:**

1. Animal Behavior and Conservation Program, Dept. of Psychology, Hunter College, City University of New York, 695 Park Avenue, Room 611N, New York, NY 10065, USA
2. Livestock and Wildlife Hospital, Faculty of Veterinary Science, Mahidol University, 199 Moo 9, Highway No. 323, Sai Yok, Kanchanaburi 71150, Thailand
3. Cognitive and Comparative Psychology Program, Graduate Center, City University of New York, 365 Fifth Avenue, New York, NY 10016, USA

**Author for Correspondence: Joshua M. Plotnik, e-mail: Joshua.Plotnik@gmail.com**

***Present Address: Akkharatchakumari Veterinary College, Walailak University, 222 Thaiburi, Thasala, Nakhon Si Thammarat 80161, Thailand**

**Keywords:** pangolins; conservation; cognition; olfactory cognition

**Supplemental Video Legends**

Video S1. In this Phase I 'olfactory' condition trial, Pluto chooses the baited container on the left side of the apparatus. Videos S1-S3 were recorded in near total darkness using the infrared recording capabilities of the SONY camera described in the Methods section.

Video S2. In this Phase II 'olfactory distance' trial, Pluto investigates both sides and chooses the baited container on the right side of the apparatus. This is a 30-cm trial.

Video S3. In this Phase II 'scent trail' trial, Pluto chooses the right trail leading to the baited food container in an F vs. W trial.
